# The Belgian Diabetes in Pregnancy Study (BEDIP-N), a multi-centric prospective cohort study on screening for diabetes in pregnancy and gestational diabetes: methodology and design

**DOI:** 10.1186/1471-2393-14-226

**Published:** 2014-07-11

**Authors:** Katrien Benhalima, Paul Van Crombrugge, Johan Verhaeghe, Sofie Vandeginste, Hilde Verlaenen, Chris Vercammen, Els Dufraimont, Christophe De Block, Yves Jacquemyn, Farah Mekahli, Katrien De Clippel, Roland Devlieger, Chantal Mathieu

**Affiliations:** 1 Department of Endocrinology and Department of Obstetrics & Gynecology, UZ Gasthuisberg, KU Leuven, Herestraat 49, 3000 Leuven, Belgium; 2 Department of Endocrinology and Department of Obstetrics & Gynecology, OLV ziekenhuis Aalst-Asse-Ninove, Moorselbaan 164, 9300 Aalst, Belgium; 3 Department of Endocrinology and Department of Obstetrics & Gynecology, OLV ziekenhuis Aalst-Asse-Ninove, Bloklaan 5, 1730 Asse, Belgium; 4 Department of Endocrinology and Department of Obstetrics & Gynecology, Imelda ziekenhuis, Imeldalaan 9, 2820 Bonheiden, Belgium; 5 Department of Endocrinology and Department of Obstetrics & Gynecology, UZA, Wilrijkstraat 10, 2560 Edegem, Belgium; 6 Department of Endocrinology and Department of Obstetrics & Gynecology, Kliniek St-Jan Brussel, Kruidtuinlaan 32, 1000 Brussel, Belgium

**Keywords:** Overt diabetes in pregnancy, Gestational diabetes, Screening, Diagnostic criteria

## Abstract

**Background:**

The International Association of Diabetes and Pregnancy Study Groups (IADPSG) recommends universal screening with a 75 g oral glucose tolerance test (OGTT) using stricter criteria for gestational diabetes (GDM). This may lead to important increases in the prevalence of GDM and associated costs, whereas the gain in health is unclear. The goal of ‘The Belgian Diabetes in Pregnancy Study’ (BEDIP-N) is to evaluate the best screening strategy for pregestational diabetes in early pregnancy and GDM in an ethnically diverse western European population. The IADPSG screening strategy will be followed, but in addition risk questionnaires and a 50 g glucose challenge test (GCT) will be performed, in order to define the most practical and most cost effective screening strategy in this population.

**Methods:**

BEDIP-N is a prospective observational cohort study in 6 centers in Belgium. The aim is to enroll 2563 pregnant women in the first trimester with a singleton pregnancy, aged 18–45 years, without known diabetes and without history of bariatric surgery. Women are universally screened for overt diabetes and GDM in the first trimester with a fasting plasma glucose and for GDM between 24–28 weeks using the 50 g GCT and independently of the result of the GCT, all women will receive a 75 g OGTT using the IADPSG criteria. Diabetes and GDM will be treated according to a standardized routine care protocol. Women with GDM, will be reevaluated three months postpartum with a 75 g OGTT. At each visit blood samples are collected, anthropometric measurements are obtained and self-administered questionnaires are completed. Recruitment began in April 2014.

**Discussion:**

This is the first large, prospective cohort study rigorously assessing the prevalence of diabetes in early pregnancy and comparing the impact of different screening strategies with the IADPSG criteria on the detection of GDM later in pregnancy.

**Trial registration:**

ClinicalTrials.gov: NCT02036619. Registered 14-1-2014.

## Background

Gestational diabetes (GDM) is a frequent medical condition during pregnancy and was historically defined as ‘any degree of glucose intolerance with onset or first recognition during pregnancy’ [1]. GDM has long been known to raise the risk of a large for gestational age (LGA) baby and macrosomia resulting in increased rates of shoulder dystocia and caesarian deliveries [2,3]. Shortly after the delivery the glucose values are generally restored to normal, but women with GDM have a seven-fold increased risk of developing type 2 diabetes (T2DM) [4]. The initial criteria for diagnosis of GDM were established more than 40 years [5]. These criteria were chosen to identify women at high risk for development of diabetes after pregnancy and not necessarily to identify pregnancies with increased risk for adverse perinatal outcome [6].

Meanwhile, two large randomized intervention trials have demonstrated improvement in perinatal outcomes in the group of women who received treatment of mild glucose intolerance during pregnancy, especially in the frequency of LGA [7,8]. Lack of international uniformity in the approach to ascertainment and diagnosis of GDM has been a major hurdle to compare the results of both studies. The ACHOIS study used a 75 g oral glucose tolerance test (OGTT) using the former WHO criteria for GDM while the study of Landon et al. used a 3-hour OGTT with the Carpenter & Coustan criteria for GDM [7,8]. Progressively more data have shown that the risk of adverse perinatal outcomes is also associated with degrees of hyperglycaemia less severe than overt diabetes during pregnancy. The ‘Hyperglycemia and Adverse Pregnancy Outcome Study’ (HAPO), showed a continuous and graded relationship between maternal hyperglycaemia and the risk for an adverse perinatal outcome, independent of other risk factors [2].

In June 2008, the ‘The International Association of Diabetes and Pregnancy Study Groups’ (IADPSG) organized a conference leading to a new consensus statement for a new screening strategy and diagnosis of GDM [9]. Women with overt diabetes are at an increased risk for congenital anomalies and diabetes complications due to their greater degree of hyperglycaemia earlier in pregnancy [10]. The IADPSG advises therefore to screen for existing but unknown diabetes at the first prenatal clinic visit, especially in high risk populations. Cut-offs for tests used to detect diabetes in the non-pregnant population are recommended in early pregnancy [fasting plasma glucose ≥ 126 mg/dl (7.0 mmol/l), random plasma glucose ≥ 200 mg/dl (11.1 mmol/l) or HbA1c ≥ 6.5% (47 mmol/mol)]. If overt diabetes or GDM has not been diagnosed in early pregnancy or enrollment is at 24 weeks gestation or later, IADPSG advises that every woman should undergo a 75 g OGTT. The cut-off values for the FPG, 1-h and 2-h OGTT were chosen to reflect an increase in risk of 75% for the development of a birth weight > 90th percentile, an umbilical cord C-peptide in the baby > 90th percentile and percentage body fat in the baby > 90th percentile. One abnormal value is now enough to diagnose GDM [FPG ≥ 92 mg/dl (5.1 mmol/l); 1-h plasma glucose ≥ 180 mg/dl (10 mmol/l); 2-h plasma glucose ≥ 153 mg/dl (8.5 mmol/l)]. By these new criteria, the total incidence of GDM in the HAPO cohort was 16.1% but with a substantial variation among sites, ranging from 8.7% in Israel and 23.7% in the US [11]. It is generally considered that there is not enough evidence to recommend screening and treatment of GDM before 24–28 weeks of gestation. IADPSG recommends now that a FPG ≥ 92 mg/dl (5.1 mmol/l) in early pregnancy be classified as GDM [9]. This was mainly reached by consensus only and uses the cut-off derived from the HAPO study in the second part of pregnancy.

Internationally, the IADPSG recommendation for screening for GDM remains controversial since this will lead to an important increase in the prevalence of GDM, workload and associated costs. For instance, in a Norwegian population with 40% from ethnic minorities, GDM prevalence would increase from 13.0% with the former WHO criteria to 31.5% with the IADPSG criteria [12]. Other raised comments are the paucity of data on the cost effectiveness of such screening strategy, the uncertainty on the clinical relevance of treatment of mild GDM based on the IADPSG criteria and the uncertainty on the risk of women who have had mild GDM to develop T2DM postpartum [13-15]. If the IADPSG would have chosen the new criteria to be based on an odds ratio of 2.0 instead of 1.75 for the development of complications in the HAPO study, the threshold for abnormal values would have been higher [FPG ≥ 95 mg/dl (5.3 mmol/l); 1-h plasma glucose ≥ 191 mg/dl (10.6 mmol/l); 2-h plasma glucose ≥ 162 mg/dl (9.0 mmol/l)] leading to a much lower GDM prevalence in the HAPO study of 8.8% compared to 16.1% with the current IADPSG criteria [9]. Another problem is that an OGTT is a poorly reproducible test [16]. The diagnosis of GDM on the basis of one abnormal value and based on only 1 test, is consequently a point of discussion since when repeating the OGTT in the same women, the test could be completely normal.

So far, two studies have addressed the cost-effectiveness of GDM screening according to the IADPSG criteria using decision analysis models for a US population. One study showed that the IADPSG recommendations are cost effective only when post-delivery care reduces diabetes incidence [17]. In the second study management based on the IADPSG criteria would be effective if treatment would result in a decreased incidence of preeclampsia with more than 0.55% and in a decreased incidence of caesarean deliveries of more than 2.7% [18].

While the ADA has since December 2010 adopted the IADPSG recommendations, the American College of Obstetricians and Gynecologists (ACOG) and an independent expert panel assigned by the National Institute of Health (NIH) continue to promote to use of the two-step screening strategy, using an universal screening strategy with the non-fasting 50 g glucose challenge test (GCT) and if abnormal followed by the 3-hour 100 g OGTT using the Carpenter & Coustan criteria or the National Diabetes and Data Group criteria [19,20] [Table 1]. The panel assigned by the NIH was particularly concerned that the adoption of the IADPSG criteria would increase the prevalence of GDM, and the corresponding costs without clear demonstration of improvements in the most clinically important health and patient-centered outcomes. Recently both the WHO and the Endocrine Society revised their guidelines and advise now to implement the IADPSG screening strategy for GDM [21,22]. The latest 2014 ADA recommendations, specify that further research is needed to establish a uniform approach to diagnosing GDM and leave now open the option between the one-step IADPSG recommendation or the two-step screening strategy as recommended by the NIH Consensus Conference [23]. Table 2 gives an overview of the different international recommendations for screening for GDM.

**Table 1:** An overview of the different diagnostic criteria for GDM

	**NDDG 3-hour 100 g OGTT**	**Carpenter & Coustan 3-hour 100 g OGTT**	**IADPSG 2-hour 75 g OGTT**
Fasting	≥105 (5.8)	≥95 (5.3)	≥92 (5.1)
1 h	≥190 (10.6)	≥180 (10.0)	≥180 (10.0)
2 h	≥165 (9.2)	≥155 (8.6)	≥153 (8.5)
3 h	≥145 (8.0)	≥140 (7.8)	
**The number of abnormal values needed for the diagnosis of GDM**	≥ 2	≥ 2	≥ 1

Values are presented in mg/dl (mmol/l). NDDG: National Diabetes Data Group; OGTT: oral glucose tolerance test; IADPSG: The International Association of Diabetes and Pregnancy Study Groups.

**Table 2:** Overview of the different international recommendations for screening for GDM

**WHO**	➢ IADPSG criteria for GDM
**Endocrine Society**	➢ One-step screening strategy with IADPSG criteria
**ADA**	Option between:
	➢ One-step screening strategy with IADPSG criteria
	Or
	➢ Two-step screening strategy with 50 g GCT and 100 g OGTT with the Carpenter & Coustan criteria or the NDDG criteria
**NIH**	➢ Two-step screening strategy with 50 g GCT and 100 g OGTT with the Carpenter & Coustan criteria or the NDDG criteria
**ACOG**	➢ Two-step screening strategy with 50 g GCT and 100 g OGTT with the Carpenter & Coustan criteria or the NDDG criteria

WHO: World Health Organization; ADA: American Diabetes Association; NIH: National Institute of Health; ACOG: American College of Obstetricians and Gynecologists; IADPSG: The International Association of Diabetes and Pregnancy Study Groups; GCT: glucose challenge test; OGTT: oral glucose tolerance test; NDDG: National Diabetes Data Group.

### The need for more research

Data from large prospective cohort studies using the IADPSG criteria in European populations are currently lacking. Moreover, there are no cost effective analysis data based on prospective studies in European populations comparing the IADPSG screening strategy with the two-step screening strategy. There are also no accurate data on the prevalence of GDM in Belgium and the current practice for screening for GDM varies across different centers [24]. The lack of consensus on screening for GDM is also apparent in Belgium. A recent Flemish consensus between endocrinologists, gynecologists and general physicians advises at this moment to continue with the two-step screening strategy while the recent consensus of the French-speaking obstetricians is to adopt the proposed IADPSG screening strategy for GDM [25,26].

Data on the impact of implementing the IADPSG criteria are currently based on retrospective analysis comparing the two-step screening strategy or the former WHO criteria with the IADPSG criteria. These studies generally show that women former classified normal and now GDM by the IADPSG criteria, have an impaired gestational outcome compared to the normal glucose tolerant (NGT) women [27,28]. We have previously shown that women prior classified normal by Carpenter and Coustan criteria and now GDM by the IADPSG criteria have increased rates of caesarean section and shoulder dystocia compared to the NGT group [29]. However, retrospective analyses comparing the former ADA screening strategy using a 100 g 3 h-OGTT with the IADPSG criteria have important limitations since comparisons were drawn on different sets of criteria (the 1-hour and 2-hour tests could therefore be higher than if a 75-g OGTT would have been used) and women identified by the IADPSG criteria as GDM but considered NGT using the Carpenter & Coustan were not treated. Moreover, women were not universally screened in early pregnancy to exclude an unknown overt diabetes. Retrospective analysis from our research group has also shown that 36.1% of GDM based on the IADPSG criteria, had a FPG meeting the threshold for GDM [30]. In contrast, using the IADPSG screening strategy in a large cohort of the United Arab Emirates, FPG independently could have avoided the OGTT in 50.6% of women [31]. This highlights the need to obtain data on GDM prevalence and data on the glucose measures that fulfill the diagnostic criteria with the IADPSG criteria in different populations as this will impact the strategy used for diagnosis of GDM.

### Objective and aims of the Belgian Diabetes in Pregnancy Study (BEDIP-N)

Since many uncertainties remain concerning the on-step IADPSG screening strategy for GDM, a large prospective multi-centric cohort study was designed. The overall objective of the Belgian Diabetes in Pregnancy Study (BEDIP-N) is to evaluate the impact of the IADPSG screening strategy on the prevalence of GDM and pregnancy outcomes in an ethnic diverse population.

Specific aims are:

➢ Aim 1: To evaluate the use of a GCT as an universal screening tool in a two-step approach with the use of the 75 g 2-hour OGTT with the IADPSG criteria only if the GCT is abnormal.

➢ Aim 2: By using a multivariable risk estimation model based on the most relevant clinical risk factors and biochemical measures for GDM, the aim is to develop a simple screening algorithm.

➢ Aim 3: To evaluate differences in GDM prevalence and pregnancy outcomes using different diagnostic criteria based on the 75 g OGTT: the IADPSG criteria, the Carpenter & Coustan criteria and threshold values if diagnostic criteria would be based on an odds ratio of 2.0.

➢ Aim 4: To explore the cost effectiveness of the one-step IADPSG screening strategy.

## Methods and Design

### Study design and setting

The BEDIP-N is a national multi-centric observational and prospective cohort study with the participation of 6 centers. The University hospital of Leuven (UZ Leuven) is the coordination center.

Women are universally screened for overt diabetes and GDM during the first trimester (recruitment <14 weeks but first screening test can be delayed until max. 16 weeks) by measuring the FPG. GDM in early pregnancy is defined as a FPG ≥100 and ≤125 mg/dl (≥5.5 and ≤6.9 mmol/l), in line with the definition of prediabetes outside pregnancy [23]. Overt diabetes in early pregnancy is defined as diabetes outside pregnancy [FPG ≥126 mg/dl (7.0 mmol/l)] [23]. The IADPSG recommendation that a FPG ≥ 92 mg/dl (5.1 mmol/l) in early pregnancy be classified as GDM was not adopted since more data are necessary on its validity. Using a higher FPG cut-off for GDM in early pregnancy will allow to evaluate the number of women with a FPG < 100 mg/dl (5.5 mmol/l) in early pregnancy to later develop GDM based on the 75 g OGTT with the IADPSG criteria.

**Figure 1:**
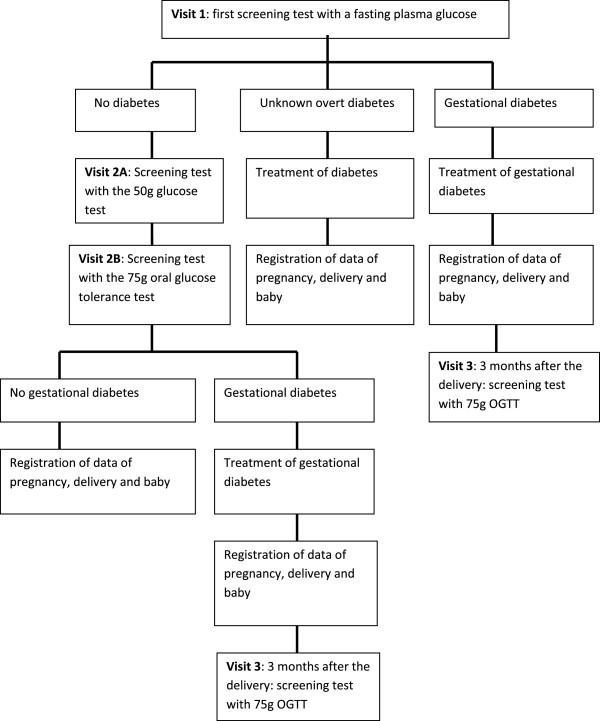
The different steps of the BEDIP-N study.

Women without diabetes or GDM in early pregnancy, will be universally screened for GDM between 24–28 weeks of pregnancy, using the 50 g GCT and independently of the result of the GCT, will all receive the 75 g OGTT. The diagnosis of GDM will be based on the 75 g OGTT with the IADPSG criteria. Participants and researchers will therefore be blinded for the result of the GCT. The result of the GCT test will later be used to evaluate the use of a GCT in a two-step approach with the use of the 75 g OGTT with the IADPSG criteria only if the GCT is abnormal. Women with GDM, will be reevaluated three months postpartum with a 75 g OGTT using non-pregnancy diagnostic criteria. Women with diabetes or GDM will be treated according to a standardized protocol in line with current routine clinical practice.Data are collected during early and late pregnancy and at delivery and for women with GDM also at three months postpartum. At each visit blood samples are collected, anthropometric measurements are obtained and self-administered questionnaires are completed. Figure 1 gives an overview of the different steps of the study.

The Belgian National Lottery provides funding for this investigator-initiated study. The study is registered in ClinicalTrials.gov as NCT02036619. The study protocol was approved by the Institutional Review Boards of all participating centers (Belgian number: B322201420693).

### Study sample

#### Study cohort

The cohort is recruited from 6 centers: UZ Leuven, the University hospital of Antwerp (UZA), Imelda hospital Bonheiden, OLV hospital Aalst-site Aalst, OLV hospital Aalst-site Asse and St Jan hospital Brussels. Five centers are situated in the Northern part of Belgium: 2 centers in the province of Flemish-Brabant, 1 center in East-Flanders and 2 centers in Antwerp. One center is situated in Brussels, the capital city of Belgium. There is a good mix between centers with an average number of women from an ethnic minority background (BME) and centers with a high percentage of women from a BME background (St Jan Hospital Brussels and OLV hospital Aalst-site Asse). UZ Leuven and St Jan Hospital Brussels have a mean number of deliveries of 2200 per year and the other centers have between 700–1000 deliveries per year.

The aim is to enroll 2563 pregnant women in the first trimester over a 2 year recruitment period (see next paragraph for power calculation). The minimum recruitment planned per center is around 175 women per year (depending on the center between 100–300 women per year) with a max. of 25% of the pregnant population that has to be recruited. Recruitment began in April 2014 and will end around October 2016.

The expected duration of the study depends on the results of the screening tests during pregnancy. If a diagnosis is made of overt diabetes in early pregnancy or if no GDM is diagnosed early or later in pregnancy, the study will end when women and the baby leave the hospital after the delivery. For these women, the max. duration of the study will be 8 months. If women are diagnosed with GDM (early or later in pregnancy), an extra visit is planned three months after the delivery. For these women the expected duration of the trial will be max. 11 months.

#### Eligibility criteria

The inclusion criteria are:

• women 18–45 years

• a singleton pregnancy

• 6–14 weeks of gestational age

• viable pregnancy

• delivery planned in the hospital where the study is performed

The exclusion criteria are:

• < 18 years or > 45 year

• multiple pregnancy

• known diabetes or treatment with metformin

• non-viable pregnancy (miscarriage)

• chronic medical condition (uncontrolled hypertension, severe heart disease, severe chronic liver disease, severe chronic kidney disease, chronic infections such as HIV and hepatitis)

• bariatric surgery

• gastro-intestinal surgery changing the absorption of glucose (e.g. Billroth II)

• a normal follow up and treatment during pregnancy will not be possible (incompliance, psychiatric problems…)

• participating in another study 90 days before the start of the study

• planned home delivery or in a center not participating in the study

#### Power calculation and statistical analyses

We aim at demonstrating a higher proportion of macrosomia (birth weight >4 Kg) in women with GDM compared to women without GDM based on the IADPSG criteria. We assume that 5% of recruited women develop overt diabetes in early pregnancy and will be excluded from the analysis. We assume that 12.4% of eligible patients will develop GDM using IADPSG criteria (value as reported in ATLANTIC-DIP study) [28]. The prevalences of macrosomia for GDM and non-GDM cases used in the calculations are also based on results reported in the ATLANTIC-DIP study [28]. We calculate that the total number of subjects needed to demonstrate a difference in proportions of pregnancy complications with 80% power and 5% significance level, is 2563. The sample size calculation is based on a two-sided Chi-square test.

##### To analyze the overall objective and aim 3

The proportion of GDM cases with 95% confidence intervals will be estimated for both 2-step and one-step screening strategies. The difference in proportions between both screening strategies will be tested using McNemar’s test for paired proportions. McNemar’s test will be used for testing differences in proportions between Carpenter & Coustan criteria and IADPSG criteria, and between IADPSG criteria and diagnostic criteria based on an odds ratio of 2.0. All analyses will be performed using all valid measurements. The chi-square test for proportions will be used for the comparison of GDM and non-GDM patients on binary or categorical variables. The Mann–Whitney U test will be used in case both groups are being compared on ordinal or continuous variables. A complete case analysis will be performed including all cases for which both GDM status and characteristic or complication are observed.

##### To analyze aim 1

Sensitivity, specificity, positive predictive value and negative predictive value will be determined for the 50 g GCT as a predictor of the result of the two-step 75 g 2-hour OGTT. The analysis will be performed using all cases with valid measurements on both tests.

##### To analyze aim 2

Logistic regression models will be used with GDM as binary response variable and predictor as explanatory variable. Predictor-effects will be presented as odds ratios with 95% confidence intervals and p-values will be reported. Besides univariable analyses, a multivariable prediction model will be built, ultimately leading to a risk score definition for predicting the risk for developing GDM. A backward selection procedure will be applied for model building. A complete case analysis is planned, implying that all observations are used in the analysis for which all model variables are observed. In case of substantial missing data in predictor variables leading to power loss in the multivariable analysis, the technique of multiple imputation for dealing with missing data will be applied.

##### To analyze for aim 4

The economic evaluation will establish the cost-utility of the one-step IADPSG screening strategy from the health care payer perspective. To calculate QALYs, utility values of relevant health states (such as perfect health, GDM, preterm birth, permanent brachial plexus injury) are derived from the literature and combined with the time spent in the health state. Clinical data (such as the risk of preterm birth, the risk of GDM) are derived from the BEDIP-N study. Cost data are collected by the centers participating in the BEDIP-N study and are also extracted from the literature. The cost-utility is calculated as the cost per QALY gained in the base case analysis. A probabilistic sensitivity analysis is conducted to explore the robustness of cost-utility results to values of input parameters. Results of the probabilistic sensitivity analysis are presented on the cost-effectiveness plane and in a cost-effectiveness acceptability curve.

A 5% significance level will be adopted for all tests and all tests will be 2-sided. All analyses will be performed using SAS software, version 9.3 of the SAS System for Windows.

### Study visits

**Figure 2:**
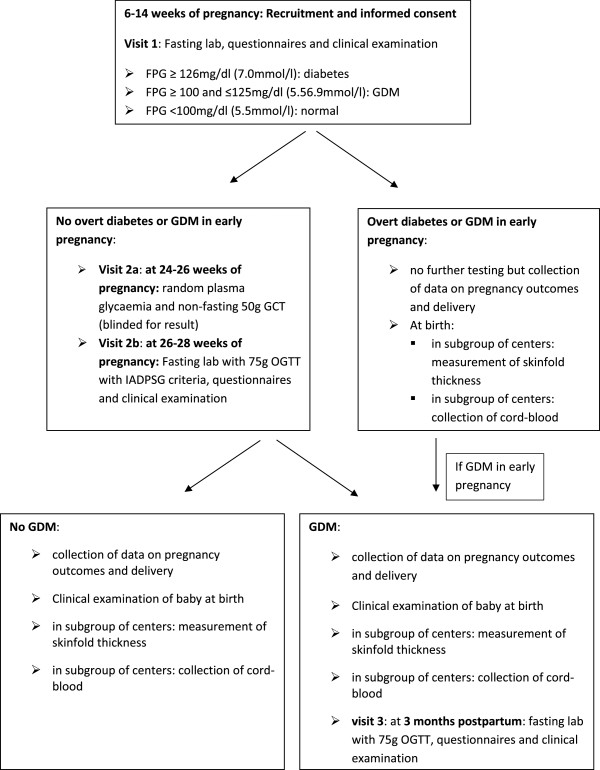
Overview of the different assessments in the BEDIP-N study.

Women are recruited by the obstetrician or midwife between 6–14 weeks of pregnancy during the first routine antenatal visit. To assess bias in recruitment, eligible women who decline participation, are asked to give a limited informed consent to collect data from the electronic medical files and to fill in a short general questionnaire on socio-economic factors. Signed informed consent for participating in the study is obtained before the first visit of the study. Informed consent includes permission for gathering data from the medical records, storage of blood samples for max. 10 years for additional analyses related to the current study and for the centers participating in the sub-studies permission for the measurement of the skin fold thickness of the baby at birth and collection of cord blood.Figure 2 gives a detailed overview the different study assessments at the different visits. Study procedures at each visit (visit 1, visit 2 and visit 3) include blood collections, anthropometric measurements and self-administered questionnaires.

#### Blood collections

Venous blood is drawn by a phlebotomist, nurse or midwife at each study visit. A fasting blood sample is drawn after a minimum of 10 hours fasting.

• For the GCT, no specific preparation is necessary. First a blood sample is collected (to later evaluate the non-fasting glycaemia), followed by a consumption of a 50 g glucose beverage (Glucomedics®) in 5 minutes and followed by a second blood sample 1 h after the intake of the glucose beverage. Data on the time of the GCT and the time of the last meal are collected.

• For the 75 g OGTT, participants are instructed to be at least 10 hours fasting and not to smoke nor engage in any physical activity during the test. They are also instructed to only drink water, but no coffee, cola or any drink containing sugar or caffeine. First, a fasting blood sample is taken, followed by a consumption of a 75 g glucose beverage (Glucomedics®) in 5 minutes. This is then followed with different blood collections at 30 minutes, 60 minutes and 120 minutes. At visit 3 women are allowed to continue breastfeeding during the OGTT and this will be recorded. The participants complete all the other study assessments during the 2-hour waiting time.

Blood samples for the analyses specifically related to the study, are in each study site processed, aliquoted and placed in a-20°C freezer within 90 min of collection. Every three months blood samples collected at the study sites are transported by a research assistant from UZ Leuven to the central laboratory of Experimental Endocrinology of the University of Leuven (KU Leuven) for longer term storage in a -80°C freezer.

#### Clinical examinations

Blood pressure (BP) is measured twice with 5 minutes interval using an automatic blood pressure monitor (Omron Philips large 34-44 cm). Height is measured to the nearest 0.5 cm using a calibrated wall-mounted stadiometer. Weight is measured using a calibrated portable Tanita HD 382 digital scale, which measures up to 150 Kg. Waist circumference is measured in centimeters by applying the tape directly on the skin, horizontally at the level laterally that is midway between the iliac crest and the lowest lateral portion of the rib cage.

At visit 1: BP, height, weight and waist circumference are measured. At visit 2b: BP and weight are measured. At visit 3: BP, weight and waist circumference are measured. BMI is calculated as weight (Kg) divided by height (m) squared.

#### Self-administered questionnaires

Table 3 gives an overview of the different questionnaires at each visit.

**Table 3:** Overview of the different self-administered questionnaires at the different visits

	**Visit 1**	**Visit 2a**	**Visit 2b**	**Visit 3**
Questionnaire on general habits and socio-economic factors	**X**			
Questionnaire on lifestyle	**X**		**X**	**X**
Questionnaire IPAQ			**X**	**X**
Questionnaire on tolerance of GCT		**X**		
Questionnaire on tolerance of OGTT			**X**	
Questionnaire on depression			**X**	**X**
Questionnaire on breastfeeding/contraception				**X**
Questionnaire on general health				**X**

IPAQ: International physical activity questionnaire; GCT: glucose challenge test; OGTT: oral glucose tolerance test.

• **Questionnaire on general habits and socio-economic factors**

• We use a self-designed questionnaire to extensively collect information on family history of diabetes, smoking, alcohol and illicit drug habits, ethnic origin and education level of the participant and her parents. We also ask about marital status, employment and income.

• **Questionnaire on lifestyle**

• We use a questionnaire on lifestyle that has been previously used to question daily walking activities and servings per weeks of different important food categories and beverages [32].

• **The international questionnaire on physical activity (IPAQ)**

• The IPAQ questionnaire is a wildly used questionnaire that is also validated for use in the Belgian population and has also been used in pregnancy [33]. IPAQ extensively questions different areas of physical activity such as job-related physical activity, transportation, house work and caring for family, recreation and time spent sitting. We have added a question on the time watching television or playing computer games to better assess sedentary behavior.

• **Questionnaires evaluating tolerance of GCT and OGTT**

• We use a self-designed questionnaire to collect information on the tolerance of the different screening tests for GDM and also ask for the preference of the participants for either test.

• **Questionnaire on depression**

• The 20-item Center for Epidemiologic Studies-Depression (CES-D) has been widely used with pregnant women and postpartum women to asses depression symptoms over the past 7 days [34].

• **Questionnaire on breastfeeding and contraception**

• We use a self-designed questionnaire to extensively collect information on the duration and frequency of breastfeeding as well as on the type of contraception used.

• **Questionnaire on General health (SF-36)**

• The SF-36 health survey is used as a questionnaire on general health and has been validated for use in the maternity context [35]. Data from this questionnaire are used to calculate Qualy’s to explore the cost effectiveness of the one-step IADPSG screening strategy. The SF-36 will be obtained at visit 3 from women with previous GDM and will be send by mail to the other participants three months postpartum.

### Outcomes of the study

#### The diagnosis of GDM

Diabetes and GDM in early pregnancy are resp. defined as diabetes and prediabetes outside pregnancy [23]. The diagnosis of GDM between 24–28 weeks of pregnancy is based on the IADPSG criteria [9]. For women with a diagnosis of diabetes or GDM, data on the treatment and glycemic control will be collected at each visit with the diabetes nurse, dietician and/or diabetologist. The following data are collected: whether glycaemic targets are met or not, when and why insulin is started and changes in the dose of insulin. All concomitant medication and/or supplements will also be recorded.

#### Pregnancy and delivery outcome data

• *Maternal data* that are prospectively collected are: pregnancy duration, preeclampsia (de novo BP ≥140/90 mmHg > 20 weeks with proteinuria or signs of end-organ dysfunction), eclampsia, HELLP syndrome (according to the Tenessee criteria) [36], gestational hypertension (de novo BP ≥140/90 mmHg > 20 weeks), preexisting hypertension, pregnancy-induced cholestasis, ultrasound data on the presence of hydramnios (amniotic fluid index >25 cm), abdominal circumference ≥ P95 and estimated fetal weight ≥ P90 or ≤ P10.

• *Delivery data* that are prospectively collected are: type of labor (spontaneous, induced or caesarean before labor) and the indications if appropriate, type of delivery (spontaneous vaginal, forceps or vacuum, caesarean section during labor or planned caesarean section) and the indications if appropriate.

• *Neonatal data* that are prospectively collected are: macrosomia (>4 Kg), LGA (birth weight >90 percentile according to standardized Flemish birth charts adjusted for sex of the baby and parity), small for gestational age (birth weight <10 percentile according to standardized Flemish birth charts adjusted for sex of the baby and parity), preterm delivery (<37 completed weeks), 1 and 5 min Apgar score, shoulder dystocia, birth trauma, neonatal respiratory distress syndrome, congenital anomalies, neonatal hypoglycaemia, neonatal jaundice, hypocalcaemia, hypomagnesemia, polycythaemia and duration and indication for admission on the neonatal intensive care unit. Neonatal blood analyses are only done if there is a clinical warning sign in accordance with local practice.

Researchers are asked to evaluate whether the main reason of the maternal and neonatal complications and management is related to diabetes or GDM.

### Other study measurements

#### Evaluation of insulin sensitivity and beta-cell function during pregnancy and postpartum based on the OGTT

At the three visits blood samples are taken to later determine HbA1c and insulin (fasting and at the different time frames of the 75 g OGTT). These analyses will be performed in all women with diabetes or GDM and in a control group of women without diabetes or GDM. Analyses will be performed at the central lab of UZ Leuven. HbA1c is measured by reversed-phase cation-exchange chromatography (ADAMS HA-8160, Menarini Diagnostics Benelux, Zaventem, Belgium). Plasma glucose is measured by an automated colorimetric-enzymatic method (hexokinase-glucose-6-phosphate-dehydrogenase, application 668) on a Hitachi/Roche-Modular P analyzer. Insulin is measured by the immunometric ECLIA (Roche Modular E170, Basel, Switserland).

Glycaemia will be assessed by the area under the glucose curve during the OGTT, calculated using the trapezoidal rule. Insulin sensitivity will be measured using the insulin sensitivity index of Matsuda and DeFronzo [37]. Beta-cell function will be assessed by the insulinogenic index dived by HOMA-IR [38]. The insulinogenic index will be calculated as the incremental change in insulin concentration during the first 30 min of the OGTT divided by the incremental change in glucose during the same period [39,40]. As a measure of beta-cell compensation, the insulin secretion sensitivity index (ISSI-2) will be measured [41,42]. All these measures are validated for use in pregnancy.

#### Measurement of lipids and inflammatory biomarkers

Based on blood samples taken at visit 1, lipids (total cholesterol, HDL, triglycerides, calculated LDL), 25-OH vitamin D and the inflammatory biomarkers adiponectin, leptin and Hs-CRP will be measured at the Lab of Experimental Endocrinology of KU Leuven in the group with diabetes/GDM and in a subgroup of women without diabetes or GDM. Lipids will also be measured at visit 2 and visit 3. Lipids are measured by the immunoassay analyzer Cobas 8000 (Roche). Hs-CRP, leptin and adiponectin will be measured by the immunoassay system of Meso Scale Discovery, Gaithersburg, USA (coefficients of variability are < 10% for CRP, < 8.4% for leptin and <6.5% for adiponectin). 25 OH vitamin D will be measured by the competitive radioimmunoassay (DiaSorin, Stillwater, Minnesota, USA) as previously described [43].

#### Measurement of the thyroid function

Since (subclinical) hypothyroidism is related to an increased risk for GDM, the thyroid function will be measured at visit 1 with TSH and total T4 and at visit 3 with TSH and free T4 [44]. Total T4 will be measured at the central lab of UZ Leuven. In UZ Leuven TSH is measured by immunometric ECLIA (Roche Modular E170, Basel, Switserland) and total T4 and free T4 by competitive ECLIA (Roche Modular E170, Basel, Switserland).

#### Measurement of antibodies according to the routine guidelines of the Belgian Diabetes Registry (BDR)

In women with diabetes or GDM, extra blood samples will be taken during their routine follow up by the diabetes team for the measurement of antibodies by the lab of UZ Brussels in accordance with the normal BDR regulations: new diagnosis of diabetes or GDM in women < 40 years. The antibodies that will be measured are: insulin antibodies (IAA), protein tyrosine phosphatase antibody IA-2A and glutamic acid decarboxylase (GAD) antibody as described previously by the BDR [45].

#### Screening for MODY-2

In women with a FPG ≥92 mg/dl (5.1 mmol/l) at visit 1 or visit 2B, analysis of the genetic mutation predisposing to MODY-2 will be performed at the medical genetics lab at UZA (MODY MASTR assay of Multiplicom with sequencing on MiSeq Illumina) in the following subgroups:

• In women at visit 1 or visit 2B with FPG ≥92 mg/dl (5.1 mmol/l) and a history of a first degree relative with diabetes/GDM or a prepregnancy BMI <25 Kg/m^2^.

• In a subgroup of women with FPG ≥92 mg/dl (5.1 mmol/l) in early or late pregnancy without family history of diabetes or with a BMI ≥25 Kg/m^2^.

### Evaluation of the newborn within 3 days after delivery

• Data from the routine clinical examination are collected: weight, length and head circumference

• In 4 participating centers (UZ Leuven, UZA, OLV-Aalst-site Asse and Imelda Bonheiden): measurement of the triceps, subscapular and flank skinfold thickness (two consecutive measurements are taken and recorded, the mean will be calculated) with a Harpender skinfold caliper for the calculation of the percentage of body fat as previously described in the HAPO study [46]. The measurements will be performed by a trained physician, nurse or midwife. Participating health care workers receive training for the skinfold measurements at UZ Leuven.

• In 5 participating centers (UZ Leuven, UZA, OLV-Aalst-site Asse, Imelda Bonheiden and St Jan hospital Brussels): cord blood will be collected both in women with and without diabetes/GDM for storage in the lab of Experimental lab of Endocrinology of KU Leuven for later analyses of C-peptide, lipids and leptin [2]. An extra sample of cord blood will also be collected for later epigenetic research. Since it is not yet defined which specific epigenetic research will be performed, a new application to the ethical committees will be done for this research.

### A standardized protocol in line with routine care for the treatment of diabetes/GDM

Within 2 weeks after the diagnosis, women will receive advice from the dietician and the diabetes nurse. A follow up visit and/or telephonic or email contact is planned every 7–14 days until the delivery with the dietician and/or diabetes nurse under the supervision of the diabetologist. If insulin is needed, a follow up visit and/or telephonic or email contact is planned every 7–14 days with the diabetologist until the delivery.

#### Dietary advice

Women will receive personal advice by a dietician. The institute of Medicine (IOM) guidelines for weight gain are followed [47].

#### Physical activity

If there are no contra-indications according to the obstetrician for the safety of the pregnancy, regular moderated physical activity, defined as 30 min physical activity five times a week, is advised.

#### Self-monitoring of blood glucose

The self-monitoring of the blood glucose will have to be performed at least 4 times daily (fasting and postprandial after three meals) during the first two weeks after diagnosis and thereafter at least four measurements per day during at least two days per week. The mean glycemic targets are a FPG < 95 mg/dl (5.3 mmmol/l) and 1-h after the meal <140 mg/dl (7.8 mmol/l) or 2 h after the meal <120 mg/dl (6.6 mmol/l). Centers are at liberty to choose whether the postprandial glucose level is measured after 1 h or 2 h, in accordance with their current practices but the blood glucose should always be measured at the same time interval in any given subject. For the study, only one type of glucometer (BGStar, Sanofi) is used in all centers as this will allow for more uniformity.

To uniform the initiation for insulin therapy, the ‘Weekly Average Glycaemia’ (WAG) will be calculated based on the self-monitoring values of the blood glucose (fasting and postprandial) during the first weeks after the diagnosis.

Therapy with Insulin will become necessary if the following targets are not reached:

• Fasting WAG ≥95 mg/dl (5.3 mmol/l) two weeks in a row (start of basal insulin)

• Postprandial WAG ≥140 mg/dl (7.8 mmol/l) (1 h) or ≥120 (6.6 mmol/l) (2 h) two weeks in a row (start of prandial insulin)

• Fasting and postprandial WAG over the target two weeks in a row (start of basal and prandial insulin)

• Due to obstetrical conditions such as polyhydramnios and macrosomia, according to the judgment of the obstetrician

• Fasting and postprandial WAG in the target with one or several particularly high glycaemia levels

Treatment with oral antidiabetics including metformin and glibenclamide are not allowed in the study.

Permitted insulins during the study are:

• The short and long acting human insulins.

• The short acting insulin analogues Lispro and Aspart.

• Glargine will not be used during the study since there is no official approval for use during pregnancy. Detemir can be used during pregnancy but this is only reimbursed for T1DM in Belgium.

### Quality control procedures

Every site is opened after a first initiation visit. During the study conduct, the coordinators of the study will conduct periodic monitoring visits of sites to ensure that the protocol is being followed. A monitoring visit for all sites will be performed at least once during the enrollment process. A research assistant of UZ Leuven will visit each study site at least every three months to collect the blood samples for transportation to the central lab of UZ Leuven and will also perform regular monitoring.

Detailed manuals were developed describing the data collection at the different visits and the handling of the blood samples. Any unanticipated (serious) adverse events (AE/SAE) occurring within start of the study is recorded by the investigator on the specific AE/SAE pages of the CRF in terms of nature of the event, date, outcome, and action taken. If the AE meets the definition of an SAE (according to the judgment of the investigator), the investigator must complete the SAE Report Form in addition. Research staff completes training led by the Principal investigator. Training for the skinfold measurements is organized during half a day at UZ Leuven.

A database in “Filemaker Pro” has been developed specifically for this study to enter all the study data. This includes all data from the different visits, prenatal and delivery outcome data and blood analyses. Data from the questionnaires are processed by “teleform” and then exported to the database. Each participant gets a subject identification number to ensure confidentiality of the data. All data collected in this study are referred to by subject identification number only. All data are stored in a secure manner through password protection.

Different strategies are in place to increase participation and retention. Posters, flyers and a website have been developed to give information to as many pregnant women as possible. To increase recruitment of women with an ethnic minority background, flyers are available in Dutch, French and English and the informed consent is available in Dutch, French, English, Arabic and Turkish. The study provides the participants, the primary care physician and the obstetrician with the results of the FPG at the first visit and with the results of the OGTT’s at the later visits. Women are allowed to breastfeed during the OGTT postpartum. Incentives for the participants include remuneration of the transportation costs by a 5 euro gift card for attending visit 1 and a 10 euro gift card for attending visit 2a. Retention is maximized by phone or email contact before each scheduled study visit. In addition, contact information is confirmed at any in-person, phone or email contact.

## Discussion

BEDIP-N is the first study involving a large European cohort that rigorously assesses diabetes and GDM in early pregnancy and universally screens for GDM later in pregnancy using both a GCT and 75 g OGTT with the IADPSG criteria. Strengths of the study are the large cohort that is recruited from both university and non-university centers with a good mix of centers with an average number of women with a BME background and centers with a high percentage of women with a BME background. This increases our chances to obtain a representative sample which will permit generalizability of the findings to the whole Belgian population.

A particular advantage of BEDIP-N is the detailed registration of many clinical and biochemical risk factors for diabetes and GDM with the first collection of data in the first trimester. Moreover very detailed data on dietary habits, physical activity and socio-economic status are collected. This is to our knowledge also the first study to evaluate the value of the IADPSG criteria in a two-step procedure with a 50 g GCT with the aim to explore the cost effectiveness of the one-step IADPSG screening strategy compared to other screening approaches (risk factor and two-step).

The BEDIP-N study also has strong potential to better elucidate the underlying pathophysiological mechanisms of GDM. The study includes an extensive evaluation of the insulin sensitivity and beta-cell function during pregnancy and postpartum based on the OGTT. The prevalence of auto-immunity and MODY-2 will also be evaluated in this cohort. BEDIP-N also plans extensive evaluation of newborns with the measurement of skinfold thickness to better evaluate the percentage of body fat and with the collection of cord blood for the evaluation of fetal hyperinsulinaemia.

Since this is an observational study, women are not randomized to a two-step versus a one-step screening strategy and all women are diagnosed and treated based on the 75 g OGTT. Another limitation is that the study is not powered to evaluate rarer complications such as shoulder dystocia and birth trauma. The follow up of the study is limited to three months postpartum for women with a previous history of GDM and the study will therefore not inform on the risk to develop diabetes and prediabetes in the long term.

Findings of the BEDIP-N study may have a significant public health impact since data from the study may translate into the development of a cost effective and simple screening algorithm for GDM. A better understanding of the underling mechanisms of the development of GDM might also guide further follow up and treatment.

## Competing interests

The authors declare that they have no competing interests.

## Authors’ contributions

KB, PVC, JV, RD and CM have made substantial contributions to the conception and the design of the study and have been involved in drafting the manuscript. SV, HV, CV, ED, CD, YJ, FM and KD have been involved in revising it critically for important intellectual content. All authors have given final approval of the version to be published.

## Pre-publication history

The pre-publication history for this paper can be accessed here:

http://www.biomedcentral.com/1471-2393/14/226/prepub

## References

[B1] American Diabetes Association Diagnosis and classification of diabetes mellitus Diabetes Care 2009 32 S62 S67 19118289 10.2337/dc09-S062 PMC2613584

[B2] Metzger BE Lowe LP Dyer AR Trimble ER Chaovarindr U Coustan DR Hadden DR McCance DR Hod M McIntyre HD Oats JJ Persson B Rogers MS Sacks DA HAPO Study Cooperative Research Group Hyperglycemia and adverse pregnancy outcomes N Engl J Med 2008 358 1991 2002 18463375 10.1056/NEJMoa0707943

[B3] Gilmartin AB Ural SH Repke JT Gestational diabetes mellitus Rev Obstet Gynecol 2008 1 129 134 19015764 PMC2582643

[B4] Bellamy L Casas JP Hingorani AD Williams D Type 2 diabetes mellitus after gestational diabetes: a systematic review and meta-analysis Lancet 2009 373 1773 1779 19465232 10.1016/S0140-6736(09)60731-5

[B5] O’Sullivan JB Mahan CM Criteria for oral glucose tolerance test in pregnancy Diabetes 1964 13 278 285 14166677

[B6] Metzger BE Buchanan TA Coustan DR de Leiva A Dunger DB Hadden DR Hod M Kitzmiller JL Kjos SL Oats JN Pettitt DJ Sacks DA Zoupas C Summary and recommendations of the Fifth International Workshop-Conference on Gestational Diabetes Mellitus Diabetes Care 2007 30 suppl 2 S251 S260 17596481 10.2337/dc07-s225

[B7] Crowther CA Hiller JE Moss JR McPhee AJ Jeffries W Robinson JS Australian Carbohydrate Intolerance study in Pregnancy Women (ACHOIS) Trial Group. Effect of treatment of gestational diabetes mellitus on pregnancy outcomes N Engl J Med 2005 352 2477 2486 15951574 10.1056/NEJMoa042973

[B8] Landon MB Spong CY Thom E Carpenter M Ramin SM Casey B Wapner RJ Varner MW Rouse DJ Thorp JM Jr Sciscione A Catalano P Harper M Saade G Lain KY Sorokin Y Peaceman AM Tolosa JE Anderson GB Eunice Kennedy Shriver National Institute of Child Health and Human Development Maternal-Fetal Medicine Units Network A multicenter, randomized trial of treatment for mild gestational diabetes N Engl J Med 2009 361 1339 1348 19797280 10.1056/NEJMoa0902430 PMC2804874

[B9] International Association of Diabetes and Pregnancy Study Groups Consensus Panel International association of diabetes and pregnancy study groups recommendations on the diagnostic and classification of hyperglycemia in pregnancy Diabetes Care 2010 33 676 682 20190296 10.2337/dc09-1848 PMC2827530

[B10] Lawrence JM Contreras R Chen W Sacks DA Trends in the prevalence of preexisting diabetes and gestational diabetes mellitus among a racially/ethnically diverse population of pregnant women, 1999–2005 Diabetes Care 2008 31 899 904 18223030 10.2337/dc07-2345

[B11] Sacks DA Hadden R Maresh M Deerochanawong C Dyer AR Metzger BE Lowe LP Coustan DR Hod M Oats JJ Persson B Trimble ER HAPO Study Cooperative Research Group Frequency of gestational diabetes mellitus at collaborating centers based on IADPSG consensus panel-recommended criteria Diabetes Care 2012 35 526 528 22355019 10.2337/dc11-1641 PMC3322716

[B12] Jenum AK Mǿrkrid K Sletner L Vangen S Torper JL Nakstad B Voldner N Rognerud-Jensen OH Berntsen S Mosdøl A Skrivarhaug T Vårdal MH Holme I Yajnik CS Birkeland KI Impact of ethnicity on gestational diabetes identified with the WHO and the modified IADPSG criteria: a population-based cohort study Eur J Endocrinol 2012 166 317 324 22108914 10.1530/EJE-11-0866 PMC3260695

[B13] Ryan EA Diagnosing gestational diabetes Diabetologia 2011 54 480 486 21203743 10.1007/s00125-010-2005-4 PMC3034033

[B14] Waugh N Pearson D Royle P Screening for hyperglycaemia in pregnancy: consensus and controversy Best Pract Res Clin Endocrinol Metab 2010 24 553 571 20832736 10.1016/j.beem.2010.06.004

[B15] Cundy T Ackermann E Ryan EA Gestational diabetes: new criteria may triple the prevalence but effect on outcomes is unclear BMJ 2014 11 348 g1567 doi:10.1136/bmj.g1567 24618099 10.1136/bmj.g1567

[B16] Harlass FE Brady K Read JA Reproducibility of the oral glucose tolerance test in pregnancy Am J Obstet Gynecol 1991 164 564 568 1992702 10.1016/s0002-9378(11)80021-9

[B17] Werner EF Petther CM Zuckerwise L Reel M Funai EF Henderson J Thung SF Screening for gestational diabetes mellitus: are the criteria proposed by the International Association of Diabetes and Pregnancy Study Groups cost-effective? Diabetes Care 2012 35 529 535 22266735 10.2337/dc11-1643 PMC3322683

[B18] Mission JF Ohno MS Cheng YW Caughey AB Gestational diabetes screening with new IADPSG guidelines: a cost-effectiveness analysis Am J Obstet Gynecol 2012 207 326e1 326e9 22840972 10.1016/j.ajog.2012.06.048 PMC4621259

[B19] Committee on Obstetric Practice Screening and diagnosis of gestational diabetes mellitus Obstet Gynecol 2011 118 751 753 21860317 10.1097/AOG.0b013e3182310cc3

[B20] Vandorsten JP Dodson WC Espeland MA Grobman WA Guise JM Mercer BM Minkoff HL Poindexter B Prosser LA Sawaya GF Scott JR Silver RM Smith L Thomas A Tita AT NIH Consensus Development Conference: Diagnosing Gestational Diabetes Mellitus NIH Consensus State Sci Statements 2013 29 1 31 23748438

[B21] The World Health Organization guideline 2013: Diagnostic criteria and classification of hyperglycaemiafirstdetectedinpregnancy [http://apps.who.int/iris/bitstream/10665/85975/1/WHO_NMH_MND_13.2_eng.pdf]. Accessed on 17-11-2013

[B22] Blumer I Hadar E Hadden DR Jovanovič L Mestman JH Murad MH Yogev Y Diabetes and pregnancy: an endocrine society clinical practice guideline JCEM 2013 98 4227 4249 24194617 10.1210/jc.2013-2465 PMC8998095

[B23] American Diabetes Association Standards of Medical Care in diabetes-2014 Diabetes Care 2014 37 S14 S80 24357209 10.2337/dc14-S014

[B24] Benhalima K Van Crombrugge P Devlieger R Verhaeghe J Verhaegen A De Catte L Mathieu C Screening for pregestational and gestational diabetes in pregnancy: a survey of obstetrical centers in the northern part of Belgium Diabetol Metab Syndr 2013 5 66 doi:10.1186/1758-5996-5-66 24405764 10.1186/1758-5996-5-66 PMC3833269

[B25] Benhalima K for the VDV-VVOG-Domus Medica consensus group The VDV-VVOG-Domus Medica consensus 2012 on screening for pregestational diabetes in pregnancy and screening for gestational diabetes P Belg Roy Acad Med 2013 2 24 42

[B26] Vanderijst J-F Debieve F Doucet F Emonts P Haumont S Hubinont C Kirkpatrick C Philips JC Pintiaux A Rousseau P Senterre G Vandeleene B Féry F Groupement des Gynécologues Obstétriciens Stratégie de dépistage et critères diagnostiques du diabète gestationnel. Propositions du GGOLFB Rev Med Liege 2012 67 4 179 185 22670444

[B27] Lapolla A Dalfra MG Ragazzi E De Cata AP Fedele D New International Association of the Diabetes and Pregnancy Study Groups (IADPSG) recommendations for diagnosing gestational diabetes compared with former criteria: a retrospective study on pregnancy outcome Diabet Med 2011 28 1074 1077 21658125 10.1111/j.1464-5491.2011.03351.x

[B28] O’Sullivan EP Avalos G O’Reilly M Dennedy MC Gaffney G Dunne F Atlantic DIP collaborators Atlantic Diabetes in Pregnancy (DIP): the prevalence and outcomes of gestational diabetes mellitus using new diagnostic criteria Diabetologia 2011 54 1670 1675 21494772 10.1007/s00125-011-2150-4

[B29] Benhalima K Hanssens M Devlieger R Verhaeghe J Mathieu C Analysis of pregnancy outcomes using the new IADPSG recommendation compared with the Carpenter and Coustan criteria in an area with a low prevalence of gestational diabetes Int J Endocrinology 2013 1 1 doi:10.1155/2013/248.121 10.1155/2013/248121 PMC3556446 23365571

[B30] Benhalima K Van Crombrugge P Hanssens M Devlieger R Verhaeghe J Mathieu C Gestational diabetes: overview of the new consensus screening strategy and diagnostic criteria Acta Clin Belg 2012 67 255 261 23019800 10.2143/ACB.67.4.2062669

[B31] Agarwal MM Dhatt GS Shah SM Gestational diabetes mellitus. Simplifying the International Association of Diabetes and Pregnancy diagnostic algorithm using fasting plasma glucose Diabetes Care 2010 33 2018 2020 20519664 10.2337/dc10-0572 PMC2928355

[B32] Duran A Matin P Runkle I Pérez N Abad R Fernández M Del Valle L Sanz MF Calle-Pascual AL Benefits of self-monitoring blood glucose in the management of new-onset type 2 diabetes mellitus: the St Carlos Study, a prospective randomized clinic-based interventional study with parallel groups J Diabetes 2010 2 203 2011 20923485 10.1111/j.1753-0407.2010.00081.x

[B33] Harrison CL Thompson RG Teede HJ Lombard CB Measuring physical activity during pregnancy Int J Behav Nutr Phys Act 2011 8 19 doi:10.1186/1479-5868-8-19 21418609 10.1186/1479-5868-8-19 PMC3069935

[B34] Dalfrà MG Nicolucci A Bisson T Bonsembiante B Lapolla A Quality of life in pregnancy and post-partum: a study in diabetic patients Qual Life Res 2012 21 291 298 21633879 10.1007/s11136-011-9940-5

[B35] Petrou S Morrell J Spiby H Assessing the empirical validity of alternative multi-attribute utility measures in the maternity context Health Qual Life Out 2009 7 40 52 10.1186/1477-7525-7-40 PMC2687423 19419553

[B36] Sibai BM The HELLP syndrome (hemolysis, elevated liver enzymes, and low platelets): much ado about nothing? Am J Obstet Gynaecol 1990 162 311 316 10.1016/0002-9378(90)90376-i 2309811

[B37] Matsuda M Defronzo RA Insulin sensitivity indices obtained from oral glucose tolerance testing. Comparison with the euglycemic insulin clamp Diabetes Care 1999 22 1462 1470 10480510 10.2337/diacare.22.9.1462

[B38] Matthews DR Hosker JP Rudenski AS Naylor BA Treacher DF Turner RC Homeostais model assessment: insulin resistance and beta-cell function from fasting plasma glucose and insulin concentrations in man Diabetologia 1985 28 412 419 3899825 10.1007/BF00280883

[B39] Kahn SE The relative contributions of insulin resistance and beta-cell dysfunction to the pathophysiology of type 2 diabetes Diabetologia 2003 43 3 19 12637977 10.1007/s00125-002-1009-0

[B40] The Diabetes Prevention Program Research Group Role of insulin secretion and sensitivity in the evolution of diabetes type 2 in the Diabetes Prevention Program: effects of lifestyle intervention and metformin Diabetes 2005 54 2404 2414 16046308 10.2337/diabetes.54.8.2404 PMC1360738

[B41] Kirwan JP Huston-Presley L Kalhan SC Catalano PM Clinically useful estimates of insulin sensitivity during pregnancy: validation studies in women with normal glucose tolerance and gestational diabetes Diabetes Care 2001 24 1602 1607 11522706 10.2337/diacare.24.9.1602

[B42] Retnakaran R Qi Y Goran MI Hamilton JK Evaluation of proposed oral disposition index measures in relation to the actual disposition index Diabet Med 2009 26 1198 1203 20002470 10.1111/j.1464-5491.2009.02841.x

[B43] Bouillon R Van HE Jans I Tan BK Van Baelen H De Moor P Two direct (nonchromatographic) assays for 25-hydroxyvitamin D Clin Chem 1984 30 1731 1736 6541534

[B44] Toulis KA Stagnaro-Green A Negro R Maternal Subclinical Hypothyroidsm and Gestational Diabetes Mellitus: A Meta Analysis Endocr Practice 2014 21 1 18 10.4158/EP13440.RA 24449677

[B45] Vermeulen I Weets I Costa O Asanghanwa M Verhaeghen K Decochez K Ruige J Casteels K Wenzlau J Hutton JC Pipeleers DG Gorus FK Belgian Diabetes Registry An important minority of prediabetic first-degree relatives of type 1 diabetic patients derives from seroconversion to persistent autoantibody positivity after 10 years of age Diabetologia 2012 55 413 420 22095238 10.1007/s00125-011-2376-1 PMC3810367

[B46] The HAPO Study Cooperative Research Group Hyperglycemia and Adverse Pregnancy Outcome (HAPO) Study. Associations with neonatal anthropometrics Diabetes 2009 58 453 459 19011170 10.2337/db08-1112 PMC2628620

[B47] Institute of Medicine (US) and National Research Council (US) Committee to Reexamine IOM Pregnancy Weight Guidelines Rasmussen KM, Yaktine AL Weight Gain During Pregnancy: Reexamining the Guidelines National Academies Press (US) 2009 Washington (DC): The National Academies Collection: Reports funded by National Institutes of Health 20669500

